# Structured-light surface scanning system to evaluate breast morphology in standing and supine positions

**DOI:** 10.1038/s41598-020-70476-2

**Published:** 2020-08-24

**Authors:** Olivia L. H. Tong, Astrid Chamson-Reig, Lawrence C. M. Yip, Muriel Brackstone, Mamadou Diop, Jeffrey J. L. Carson

**Affiliations:** 1 Imaging Program, Lawson Health Research Institute, 268 Grosvenor Street, London, ON N6A 4V2 Canada; 2 School of Biomedical Engineering, Western University, 1151 Richmond Street, London, ON N6A 3K7 Canada; 3 Department of Medical Biophysics, Western University, London, Canada; 4 London Regional Cancer Program, London Health Sciences Centre, 800 Commissioners Road East, London, N6A 5W9 Canada; 5 Department of Surgery, Western University, London, Canada

**Keywords:** Translational research, Biomedical engineering, Three-dimensional imaging, Optical metrology, Breast cancer

## Abstract

Breast shapes are affected by gravitational loads and deformities. Measurements obtained in the standing position may not correlate well with measurements in the supine position, which is more representative of patient position during breast surgery. A dual color 3D surface imaging system capable of scanning patients in both supine and standing positions was developed to evaluate the effect of changes in body posture on breast morphology. The system was evaluated with  breast phantoms to assess accuracy, then tested on ten subjects in three body postures to assess its effectiveness as a clinical tool. The accuracy of the system was within 0.4 mm on average across the model. For the human study, there was no effect of body posture on breast volumes (*p* value > 0.05), but we observed an effect of completeness of breast scans on body posture (*p* value  < 0.05). Post-hoc tests showed that the supine position and the standing position with hands at the waist differed significantly (*p* value  < 0.05). This study shows that the system can quantitatively evaluate the effect of subject postures, and thereby has the potential to be used to investigate peri-operative changes in breast morphology.

## Introduction

### Background

In conventional practice, surgeons plan breast surgeries by consulting radiographic images such as two-dimensional mammograms and breast MRI images to identify tumour locations within the breast. However, these radiographic images are taken in either the standing or prone position and do not represent the breast position during surgery. This discrepancy is further exacerbated by breast compression during mammography and breast elongation during MRI^1^. As a result, surgeons need to mentally transform the radiographic images to information that matches the surgical scenario in the supine position. This is particularly challenging for oncoplastic and reconstructive surgeries because the technique used for reconstruction depends on the tumour location and the tumour to breast size ratio^2^. Therefore, the technical challenges of anticipating tumour location in order to completely remove it and reconstruct the breast affect the success of such surgeries and leads to variable cosmetic results and patient satisfaction.

Several studies have suggested that optical three-dimensional structured illumination (3D-SI) technologies can be applied to plan and assess the outcome of oncoplastic, reconstructive, and aesthetic breast surgery^1, 3, 4^. Optical techniques capture surface information non-invasively without the use of ionizing radiation. These techniques improve safety and enhance patient comfort. Commercially available breast surface scanning systems utilize photogrammetry. For example, the Vectra XT scanner (Canfield Scientific, Fairfield, NJ, USA) has a geometric accuracy of 1 mm and an acquisition speed of 3.5 ms. Another example is the 3dMD Torso system (3dMD, Atlanta, Georgia, USA), which has a geometric accuracy of 0.2–0.5 mm and an acquisition speed of 1.5 ms^5^. Both scanners acquire data while the patient is standing. Standing is the standard position for subjective assessment of breast aesthetics; however, several studies have shown that scanning patients in the standing position results in incomplete scans that miss the inferior aspects of breasts in large-breasted women or women with ptosis. This is because large-breasted women tend to have larger inframammary folds and some areas become obscured from view when the individuals are in a standing position. As a result, the undersides of the breasts are hidden from the scanner and the scans are incomplete^6–8^. Studies have also shown that breast volume tends to be underestimated for women with large ptotic breasts because it is difficult to identify breast boundaries consistently^6^. Breasts do not have well-defined edges, and volume calculations require assumptions on the depth and curvature of the underlying chest wall^9^. This is further complicated by the fact that breast morphology is dependent on patient position. For example, Reece et al.^8^ reported that measured distances between fiducial markers on the breast surface and nipple by the 3dMD Torso system varied with subject position, and the calculated breast volumes were different between standing and supine positions^8^. The apparent change in breast volume appeared to be due to the movement of the breast tissue superiorly towards the clavicle and posteriorly towards and into the axilla during the switch from standing to supine position^8^.

As these conventional 3D scanning systems are immovable workstations, there has been interest in using handheld structured-light scanners to objectively assess breast morphology. Examples of handheld scanning systems are the Artec Eva Scanner (Artec 3D Inc., Luxembourg, Luxembourg), the iSense (3D Systems, Rock Hill, SC, USA), and the Kinect Recording System (Microsoft, Redmond, WA, USA)^10–16^. Although it is convenient to use handheld devices for breast surface imaging, validation studies reported measurement errors of 2.5 mm and breast volume error of 10%^14, 17^. Handheld systems have a smaller field of view, and clinicians need to hold the unit and manipulate around the breast surface of the patients in close proximity. As a result, patients may find this intrusive. Moreover, studies have reported involuntary breathing movements during the imaging procedure and change in posture could lead to errors in reconstruction^16, 18^. Considering the above limitations and concerns with current technology, handheld systems are still not ideal for evaluating breast morphology in different postures.

### Motivation

#### Statement of the problem

Most of the currently available commercial 3D-SI systems evaluate breasts in the standing position; however, breast shape is affected by gravitational loads and deformity. Thus, measurements obtained in the standing position may not correlate well with similar measures captured during surgery. Clinicians currently use subjective parameters obtained in the standing position to plan and assess breast reconstruction surgeries, and patient satisfaction of the procedure varies. Given the dependence of breast measures on subject posture, there is a need for a 3D-SI system capable of measuring breasts of subjects in both the supine and standing positions, which are postures used during surgical planning and surgical outcome assessment.

#### Objective

The objective of this work was to develop a 3D-SI system capable of accurately capturing the breast morphology in a single breath-hold in both the standing and supine postures. Building upon the available literature, we focused our efforts on the following developments. (1) The system should have enough coverage to measure a wide range of breast sizes, including women with ptosis. (2) The system should be able to measure the 3D surfaces of both breasts when subjects are in either the standing or supine positions. (3) The system should be a workstation that can be temporarily wheeled into the breast care clinic for assessments and then wheeled out to avoid over-crowding. In short, the system should demonstrate capabilities of assessing the effect of body posture on breast morphology quickly and quantitatively in a clinical setting.

#### Approach

In this paper, we introduce a wheeled 3D-SI system for breast assessment that used a structured-light system to provide good breast surface coverage. The 3D-SI system was mounted on an articulating arm so that the operator can easily switch the 3D-SI system to either the standing or supine positions. The system was attached to a medical cart to minimize disruption to clinical workspaces and promote clinical integration. The scanner was tested on a breast phantom to evaluate accuracy, and then on ten subjects to assess its effectiveness as a clinical tool. System performance was examined by (1) completeness and accuracy of surface scans, and (2) volume extracted from breast scans captured in various postures.

## Results

**Figure 1:**
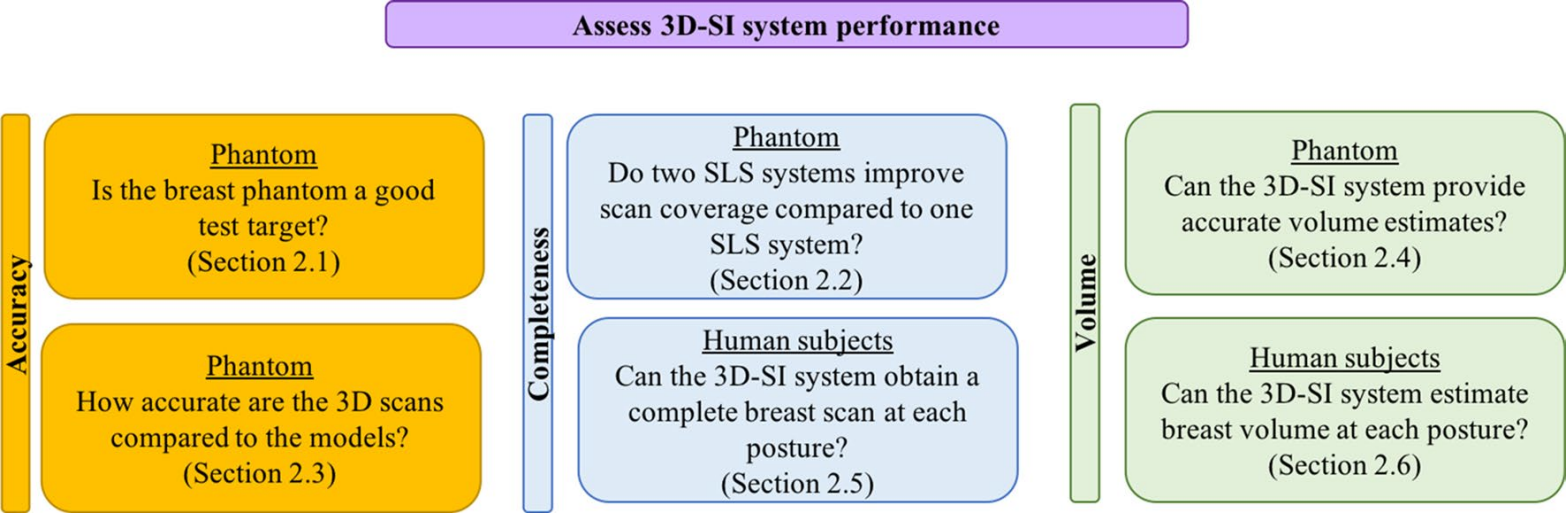
Summary of assessment of the 3D-SI system performance.

The 3D-SI system was first tested on the 3D printed breast phantoms, and then on ten human subjects. The following subsections describe procedures conducted to evaluate system performance, and the motivations for each assessment are summarized in Fig. 1.

### Assessment of print quality of the breast phantom

**Figure 2:**
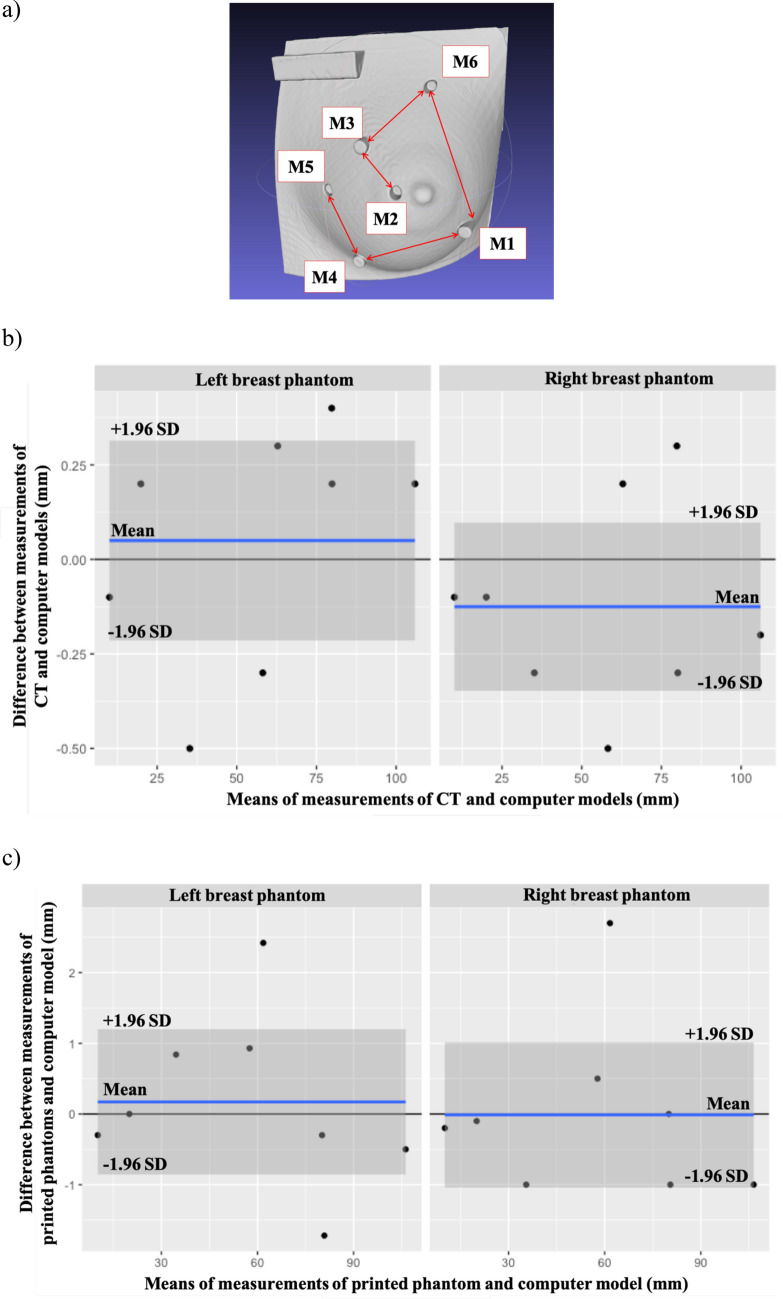
Assessment of print quality: (**a**) Reconstructed CT model. M1, M2, M3, M4, M5, and M6: Fiducial markers. Arrows are distance between the two markers. (**b**) Bland–Altman plot of the CT measurements and computer models. (**c**) Bland–Altman plot of printed phantom and computer model measurements.

The print quality of the breast phantom was evaluated based on the differences between (1) the measured values in the computed tomography (CT) reconstructed model and the computer model, and (2) the caliper measurements and the computer model. The measurements obtained were the length and width of each calibration bar, the diameter of the markers, and the distances between different markers (Fig. 2a). All the measurements are presented on Bland–Altman plots (Fig. 2b,c). There were a few outliers in both left and right breast phantoms, and the variations in CT were less than the measured values by caliper.

### Assessment of surface scan coverage on phantom

**Figure 3:**
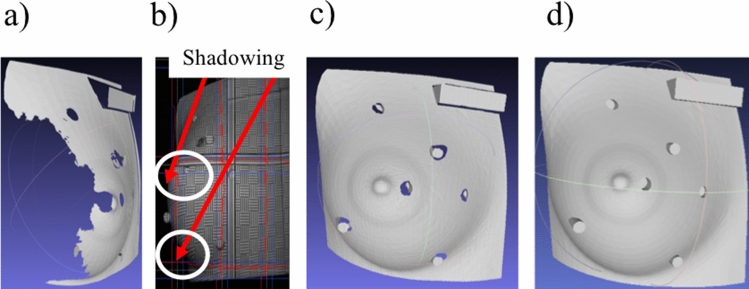
Representative scan results of the right breast phantom for supine position: (**a**) 3D scan obtained with the structured light scanner with the blue filter set. (**b**) Photograph of the projected static patterns from the blue filter set, where darker regions indicate shadowing (circled areas). (**c**) 3D scan obtained by combining the scans from the two SLS systems sequentially. (**d**) 3D scan obtained by combining the scans captured simultaneously using the dual color SLS system. The calibration bar in the top right corner of (**a**–**c**) is 80 mm long and 20 mm wide.

The 3D printed breast mimicking phantom was preliminarily scanned by one SLS system. Holes were seen in the lateral side of the polygonal meshes (Fig. 3a), and these holes corresponded to the two circled regions at the side of the breast phantom (Fig. 3b). The empty regions were areas that light could not reach, and information was missed; we termed this phenomenon shadowing. When a second projector that projected patterns at a different angle was used, areas that could not be reached by the first projector were illuminated. Figure 3c shows the merged results by combining scans from the two SLS systems sequentially, and Fig. 3d shows the merged scans taken with two SLS systems operated simultaneously.

### Assessment of accuracy of phantom surface scans

**Figure 4:**
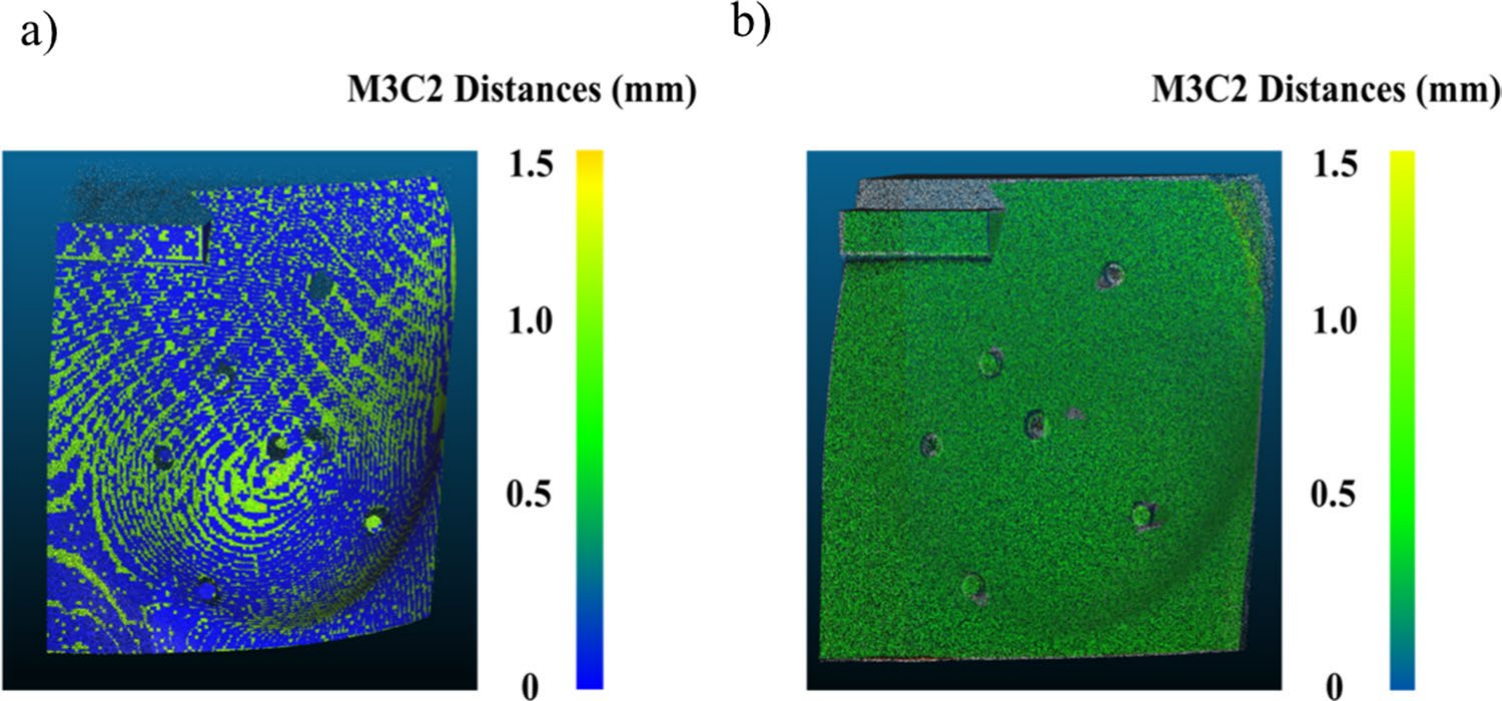
Accuracy of scanned results: (**a**) Mean distance between the surface scan and the reconstructed CT model, and (**b**) mean distance between the surface scan and the CAD model of the phantom computed by the M3C2 method.

The accuracy of the system was assessed by comparing vertex locations of the surface scans to the ones in the original CAD model. Mean distances between scan and model were assessed by multiscale model to model cloud (M3C2) algorithms. Table 1 shows the mean distances obtained by comparing the surface scans to the reconstructed CT model and CAD model. The mean distances of the CT model to surface scans accounted for the print error, and these distances were used to report the accuracy of the system. For both standing and supine positions, the vertex locations of the surface scans deviated 0.4 ± 0.7 mm on average from the original CAD model. Figure 4a,b present the surface comparison maps of the scans against the reconstructed CT model and CAD model respectively.

**Table 1:** Mean distances of surface scans.

Model	Mean distance ± RMSE* (mm)			
	CT model to surface scans		CAD model to surface scans	
	Standing	Supine	Standing	Supine
Left breast phantom	0.4 ± 0.6	0.4 ± 0.8	0.1 ± 0.7	0.1 ± 0.6
Right breast phantom	0.3 ± 0.8	0.3 ± 0.5	0.1 ± 0.3	0.0 ± 0.3

*RMSE root mean square error.

### Assessment of accuracy of estimated volumes on phantom

The calculated volume of the phantom CAD model was 3,730 cm^3^ and the volume of the phantom CT model was 3,729 cm^3^. The mean volumes from the 3D scans were 3,727 ± 12 cm^3^ and 3,732 ± 11 cm^3^ for supine and standing, respectively. The 3D scan mean volumes were 2 cm^3^ (0.05%) smaller than the CT model measurements for the supine position and 3 cm^3^ (0.08%) greater for the standing position.

### Assessment of completeness of surface scans of human participants

**Figure 5:**
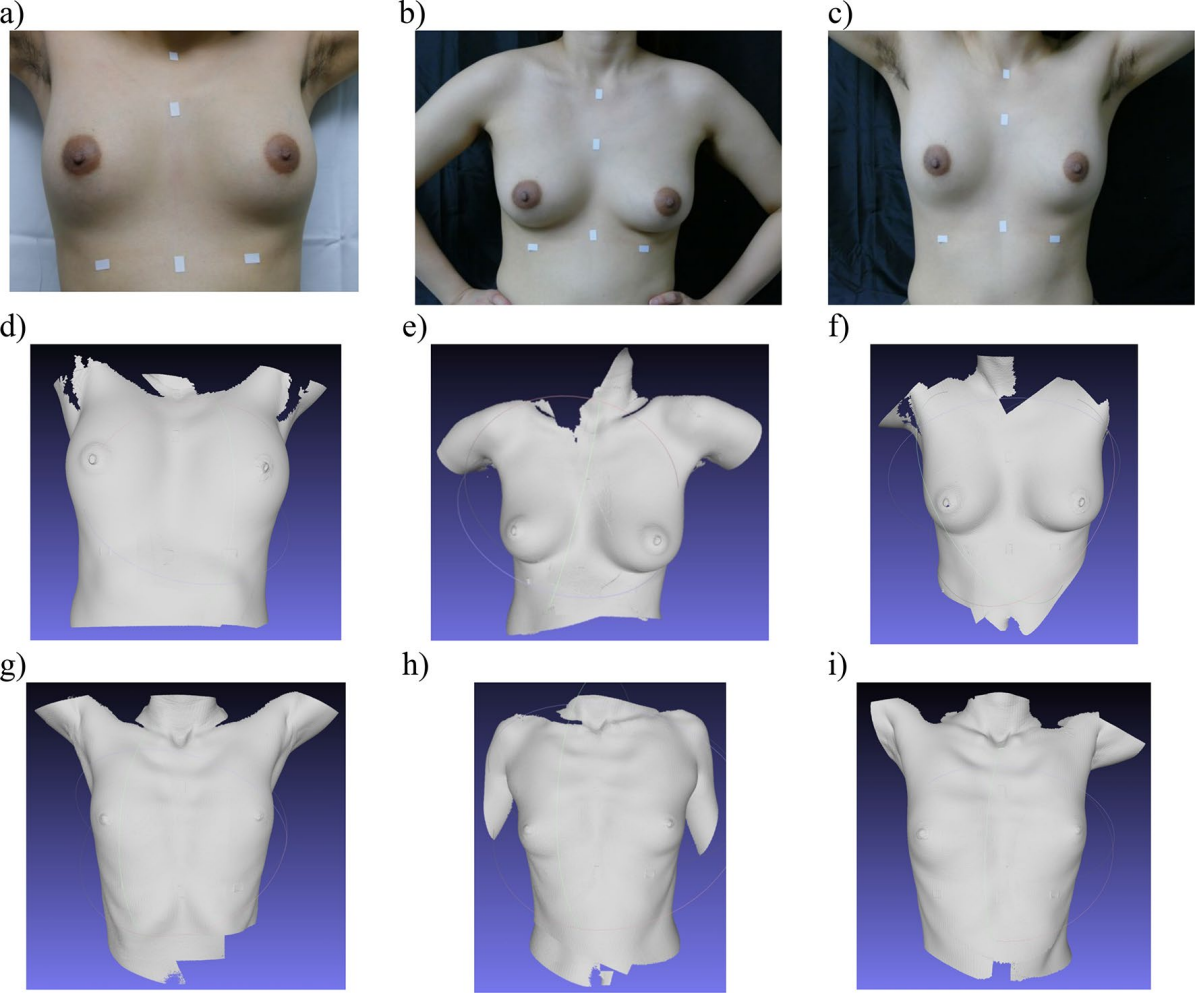
Breast surface scanning of human subjects in three postures. (**a**) Photograph of the human participant resting on a hospital bed in supine posture with both hands on their head. (**b**) Photograph of same human participant in standing posture with hands at their waist and arms in slight abduction, and (**c**) photograph of same human participant in standing position with hands behind their head. Corresponding surface maps of participant in (**d**) supine posture, (**e**) standing posture with hands at their waist and in slight abduction, and (**f**) standing posture with hands behind their head. Surface maps of another subject in (**g**) supine posture, (**h**) standing posture with hands at the waist, and (**i**) standing posture with hands behind head.

**Figure 6:**
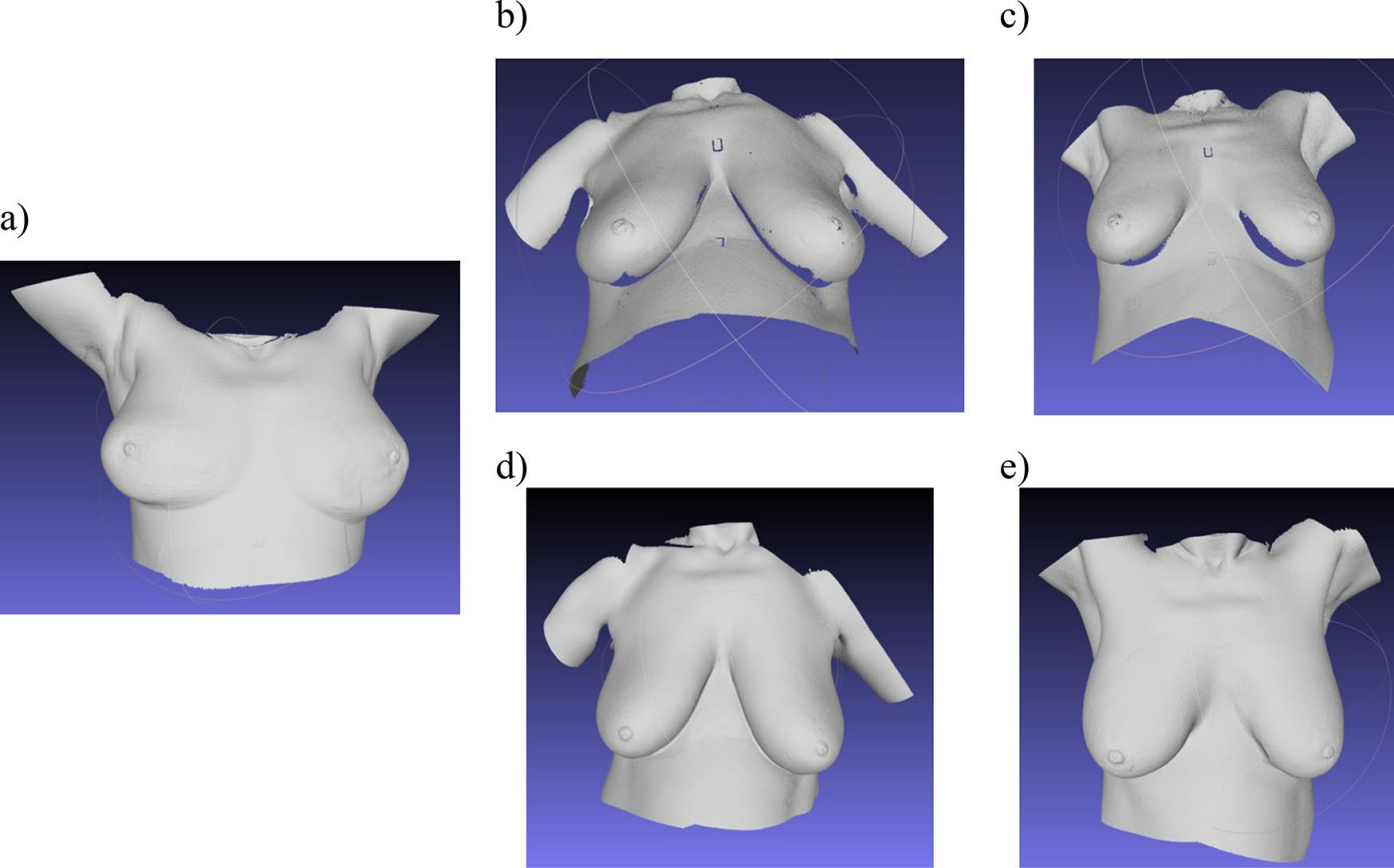
Breast surface scans of Subject 3 in supine and standing postures. A collection of 3D surface scans taken with dual color 3D-SI system and participant in the (**a**) supine posture, (**b**) standing posture with hands at their waist, and (**c**) standing posture with hands behind the head. Missing areas of the 3D surface scans were digitally filled for scans of participant in (**d**) the standing posture with hands at the waist, and (**e**) the standing posture with hands behind the head.

Breast surface scans captured from human participants, shown in Fig. 5, were acquired in a single breath-hold at each posture. The completeness of breast visualization for each of the ten participants is shown in Table 2. Examples of complete visualization are presented in Fig. 5. Breast surface scans for some individuals with bra sizes of C and D were incomplete in the standing positions as a result of shadowing (Fig. 6).

**Table 2:** Completeness of breast surface map for the three scanning positions.

Subject	Bra size	Postures		
		Supine	Standing	
		Head	Waist	Head
1	34B	Complete	Complete	Complete
2	42D	Complete	Incomplete	Incomplete
3	36D	Complete	Incomplete	Incomplete
4	30A	Complete	Complete	Complete
5	38A	Complete	Complete	Complete
6	36AA	Complete	Complete	Complete
7	32AA	Complete	Complete	Complete
8	36C	Complete	Incomplete	Complete
9	30A	Complete	Complete	Complete
10	40C	Complete	Incomplete	Complete

### Estimated volumes of human participants

For the human subject results, the estimated breast volumes at different postures are shown in Table 3. Breast volumes were in good agreement for all three positions for individuals with smaller breasts and complete breast scans. However, breast volumes varied for individuals in standing postures with incomplete breast scans. A one-way ANOVA was conducted to compare the effect of body postures on breast volumes in supine, standing with hands at the waist, and standing with hands behind the head positions. There was no effect of body posture on the breast volumes (*F *(*2, 57)* = *0.151, p* = *0.860*). A second one-way ANOVA was conducted to compare the effect of body postures on completeness of breast scans. We observed a significant effect of completeness of breast scans on body posture (*F *(*2, 57*) = *5.7, p* < *0.01*). A post hoc Tukey’s HSD test showed that the supine and the standing with hands at the waist positions differed significantly at *p* < *0.01*; all other comparisons were not significant (ps > 0.1).

**Table 3:** Breast volumes of human subjects in three scanning positions.

Subject postures		Supine		Standing			
		Head		Waist		Head	
Breast		Left	Right	Left	Right	Left	Right
Subject	Bra size	Breast volumes (cm^3^)					
1	34B	325.9	304.1	343.6	311.7	320.4	309.6
2	42D	596.7	414.7	644.1*	515.5*	602.4*	530.9*
3	36D	583.0	550.9	599.2*	590.9*	505.0*	531.9*
4	30A	98.5	64.3	97.3	59.5	93.4	52.1
5	38A	260.8	232.8	260.7	240.6	264.1	248.2
6	36AA	170.5	144.7	175.2	150.5	177.5	141.9
7	32AA	119.3	108.5	129.9	107.5	132.5	103.8
8	36C	502.5	462.6	586.9*	582.5*	511.1	474.2
9	30A	88.6	106.3	83.8	114.7	85.7	113.1
10	40C	444.2	433.8	572.8*	461.3*	449.5	425.8

*Incomplete breast scans, the undersides of the breast were missed.

## Discussion

### Major findings

#### Enhanced surface coverage with dual color 3D-SI

We have developed a dual color 3D-SI system for simultaneous 3D surface scanning to enhance surface coverage without sacrificing speed. When combined, the surface maps from the two SLS systems did not suffer from shadowing compared to a single SLS surface map of the breast phantom. We also expanded the FOV of each SLS system using cameras with larger sensors. When the system was angled at 20°, our dual color 3D-SI system enabled greater coverage and the ability to visualize curved surfaces of the breast. Our work is consistent with the work of other researchers. Notably, Kovacs et al.^19^ connected two scanners and the scanners were tilted 10° below the horizontal plane to obtain precise breast surface scans sequentially. Instead of sequential scanning, our system provided surface scans from both scanners simultaneously. The modular, multispectral nature of our system provided the possibility to add additional surface scanners to increase coverage without sacrificing speed.

#### Reduced interference during dual color 3D-SI

Each SLS system was installed with either blue or green filters. Blue and green filters were selected based on the reflectance of human skin. The chromophores in the skin, especially melanin, determine the reflectivity of skin at different wavelengths^20^. Shorter wavelengths (blue and green) are known to exhibit fewer differences in reflected light for different skin colors compared to longer wavelengths (red and orange)^21^. As a result, shorter wavelengths were chosen for the dual color 3D-SI system as less adjustment was needed. The introduction of color filters eliminated interference between the two SLS systems, and this was evident from the merged surface scans taken with the two SLS systems in Fig. 3c,d.

#### Clinical suitability

We mounted our dual color 3D-SI system onto a wheeled medical cart for use in a clinical exam room. The wheeled medical cart fit well into the tight confines of the room. The articulating arm allowed a multitude of positions and orientations of the scan head to obtain good surface coverage. The scan head could also be rotated to provide scan coverage for positions other than standing and supine, such as a sitting position, without the need for recalibration between position changes. Therefore, the system could be used by clinicians to study the effect of various body postures on breast morphologies.

#### System reliability

We evaluated the print quality of the phantom by comparing measurements of the printed phantoms and reconstructed CT models to the ones of the CAD model. The Bland–Altman plots indicated there were variations in different regions of the phantom, and the variations for caliper measurements were larger than the values obtained with the CT models. In general, the measured values of the CT and CAD models were in good agreement and the print quality was acceptable. The M3C2 algorithm was used to estimate the mean distance between the 3D scan and the model. Some of the scans of the phantom had missing areas behind the calibration bar and the protruding fiducial markers due to shadowing. The M3C2 algorithm could not properly account for these errors; it did not perform distance calculations for regions where data was missing in the scan. Another factor that affected the accuracy of the mean distance estimates was the scan-model registration error. The M3C2 algorithm accounted for this error in the uncertainties. We compared the results of the 3D-SI system to the reconstructed CT model and the computer model used to print the phantom. The discrepancy between the results of the CT model and the computer model suggested the variations in the print quality were substantial, and the CT model should be used as the reference model to account for print error. Therefore, we concluded that the accuracy of our system was within 0.4 mm on average across the model. Local variations were larger, and some areas had a difference of up to 0.8 mm. In future, the protruding markers could be removed from the 3D printed phantom to avoid scan artifacts. Overall, the accuracy of the system compared well to the performance of commercial systems which have accuracy in the millimeter range^5, 8^.

#### Completeness of surface scans

We first tested our system on a breast phantom; however, the 3D printed breast phantom had limitations as it was a rigid structure with a fixed breast size and shape regardless of posture. Therefore, we tested the system on ten human participants to provide a more realistic assessment. The system provided complete breast visualization for participants with smaller breasts (i.e. bra size of A and B) in all positions as shown in Table 2. For individuals with bra size of C, complete breast scans were obtained in the standing posture with hands behind their head, but not when the subjects placed their hands at their waist. The system also failed to obtain complete scans for the subjects with a bra size of D in all standing postures. Shadowing occurred in the standing posture and the inferior aspects of the breast could not be visualized (Fig. 6b,c). As a result, those scans were incomplete. This is consistent with the findings of Coltman et al.^22^ where full visualization was achieved in only 5% of large-breasted subjects in the standing position.

#### Estimated breast volumes

The performance of the system was also assessed by accuracy of the volumetric measurements. We demonstrated that the system had a difference of only 0.1% for the CAD-based volume analysis method. The posterior wall was created to simulate the use of a posterior breast wall for human data, and our results show that the computer-generated posterior breast wall resulted in a 0.1% volume difference between measurements made with the CT model and the CAD model. Assessment of the literature suggests that the accuracy of current volume analysis methods range from 1.1% by MRI to 8.0% by thermoplastic casting^5^. Our relative difference of 0.1% could be considered negligible compared to these current methods. This finding also increases our confidence that the system will provide reliable scans.

Further, we demonstrated the feasibility of the proposed volume analysis method by applying it to human data, where complete surface scans were acquired. Segmented breast volumes were presented, and in general, the volumes correlated well for all three positions (Table 3). For individuals with larger breasts, the segmented breast volumes measured at the supine position were smaller than the segmented volumes measured in the standing position with hands at the waist, where surface scans were incomplete. These findings supported the results of Reece et al.^8^, who obtained larger breast volumes for the standing position compared to the supine position for all five of their subjects regardless of breast size^8^. Therefore, these preliminary results suggest that the system can quantitatively evaluate changes in breast morphology resulting from changes in subject posture.

#### Standardized body position

For the standing posture, the human breast scans were collected at two different hand positions. Using an earlier iteration of this system, we initially obtained incomplete scans for all individuals with hands at the waist, while complete scans were obtained for individuals with smaller breasts with hands behind the head. We also found a significant effect of body postures on completeness of breast scans. In particular, there was a significant difference between supine and standing with hands at the waist positions. On the other hand, there was no effect of body postures on breast volume; breast volumes were not affected by the standing positions when completeness of breast scans was not considered. These findings suggested that hand position might have an influence on breast visualization. After improving the system to its current format, we obtained complete scans for smaller breasted individuals in all positions. However, we still struggled to acquire complete scans for individuals with large breasts regardless of hand position due to shadowing. Currently, there is no consensus in the literature for hand position during scans and results vary among research groups. For example, Kovacs et al.^19^ scanned 5 women in two positions, arms crossed behind the back and behind the head, and they found higher accuracy for scans when the individuals placed their arms behind the back. On the other hand, Kawale et al.^23^ scanned 12 women in two different poses: hands on the hips and hands straight down, and they found no significant differences between the two poses. These inconsistencies may have contributed to reported errors in breast volumes found in the literature for individuals with large ptotic breasts.

We obtained complete breast surface scans for all individuals regardless of breast size in the supine position. Moreover, the segmented breast volumes for individuals with smaller breasts correlated well for all three positions. For women with large breasts, our results suggest individuals should be scanned in both supine and standing positions to obtain a better assessment. While scans taken in the standing positions can evaluate cosmetic outcomes of the breast, scans taken in the supine position will provide a better quantitative assessment. Instead of digitally filling in the missing areas of the breast for breast volume analysis, our work highlights the importance of standardizing scanning methodologies, such as subject posture, to obtain more accurate and precise results.

#### 3D-SI breast scanning

The results of the study show that a 3D-SI system could be used to examine differences in breast morphologies for various postures. To the best of our knowledge, only one other group has constructed a transportable stationary 3D-SI system to investigate changes in breast morphology^8^. They mounted a photogrammetric 3D imaging (3dMDTorso) system onto a bariatric tilt table (207 cm × 79 cm × 88 cm) with the human subject tilted to various angles for investigation^8^. Our system can scan patients in more natural positions without the extra equipment needed to tilt the subjects; the system is also transportable and it can provide clinicians with images at the bedside immediately. Moreover, the dual-color 3D-SI system can fit well into tight areas around the hospital. Yip et al.^7^ first proposed the necessity of scanning large breasted women in the supine position and their findings were supported by Reece et al. and Coltman et al.^7, 8, 22^. However, current commercial scanners are still designed to image women in the standing position. As our dual-color 3D-SI system can scan patients in various positions easily, we believe this system can promote research in breast morphology and how it is influenced by different postures.

### Limitations

The major limitation of the dual-color 3D-SI system was acquisition speed. The current set-up requires 5–7 s to acquire data, and this was slower than currently available commercial systems. However, the acquisition speed of our system can be further improved by software enhancements and the use of single-shot structured light patterns at the expense of resolution and accuracy. In addition, it was estimated that post-processing of surface scans (i.e. stitching and breast volume extraction) required approximately 1 h to complete. Purpose-built software could significantly reduce post-processing processing time. With technological enhancements, it will be possible to achieve sub-second acquisition speeds with reasonable processing time comparable to commercial breast scanning systems for clinical applications.

### Future work

In the future, a larger clinical study to measure system performance across a larger cohort of human subjects could be performed. A feasibility study that scans patients in both supine and standing positions before and after breast surgery could provide useful data for determining system performance across a range of breast sizes and shapes representative of most females. This information could help plan future clinical studies and evaluate surgical techniques for breast surgeries. Moreover, scanning patients in both supine and standing positions will allow evaluation of the size and pattern of differences between the two positions, which could be used to develop models for predicting breast shape in the supine position when data is acquired in the standing position.

## Conclusion

We constructed a dual-color 3D-SI clinical system for quantitative evaluation of breast morphology for various postures. This study showed that the system can measure breast shape to evaluate the effect of subject posture. The multispectral, modular nature of the 3D-SI system enables scan coverage to be increased incrementally with additional SLS systems, thereby accommodating larger breast sizes. Since the 3D-SI system has been designed for clinical use, it could impact surgical planning and outcome assessments, and potentially improve patient satisfaction after reconstructive surgery.

## Methods

### Surface scanning of phantoms

The main hardware components of the 3D-SI system were two modified structured-light scanners (HP 3D Structured Light Scanner Pro S3, HP Inc, Palo Alto, CA, USA) mounted on a cart. Optical filters were installed on each structured-light scanning (SLS) system to minimize the interference between scanners (see Supplementary S1.1 and S1.2 for details). Data were acquired using the SLS software supplied by the manufacturer (HP 3D Scan Pro 5.4.0, HP Inc). Eighteen horizontal phase shift patterns were projected onto the object surface. Full coverage of the breast phantom was found at a working distance of 60 cm with separation of the two SLS systems by 45 cm and tilting one SLS system by 20° from vertical. The phantom of the left breast was then positioned with the nipple 60 cm away from the scanning system. The set-up was tilted to allow better coverage of the projected light and of the camera views of the breast. The system was adjusted, so that the static pattern as shown in Supplementary Fig. S2b aligned with markers on the phantom to improve consistency and reproducibility between experiments. A similar procedure was used for the phantom of the right breast. Data for a complete surface model took approximately 4–8 s to acquire. The system was then oriented for use in the standing position with a similar configuration. After collecting the patterned images, the results from each SLS system were processed in software and reconstructed into the 3D shape of the object. The surface models were then saved and exported for post-processing.

### CT imaging of phantoms

3D printed breast mimicking phantoms were imaged with x-ray CT (100 kVp, 600 mAs, 0.625 mm slice thickness, medium filter, Revolution CT, GE Healthcare, Chicago, ILL, USA). Radiographic DICOM images of the breast phantoms were then imported into an image processing program (3D Slicer 4.10, BSD licenses)^24^. Segmentation was aided by the *Threshold* tool, *Crop* tool*,* and *Segmentation editor* tool within 3D Slicer 4.10. The segmented results of left and right breast phantoms were then converted into 3D volumetric models by the *Show 3D* tool within 3D Slicer.

### Surface scanning of human participants

Human research ethics approval was obtained from both the Western University Research Ethics Board (Project ID: 110468) and Lawson Health Research Institute (R-18-471). All research was performed in accordance with the guidelines and regulations. The study was conducted in a clinical research room at the Lawson Clinical Research and Chronic Disease Centre (St. Joseph’s Hospital, London, Canada). All participants were assured that their participation was completely voluntary, and health care treatment was unaffected by participation. Written informed consent was obtained from all participants.

The 3D-SI system was used to obtain 3D surface scans of both breasts of the participants in two body postures. Five white stickers were placed on the midline and the abdomen of the participants. For the supine position, the individual was instructed to place both hands behind their head to ensure their entire breast was exposed. For the standing position, the participants were standing with their back leaning against a wall. Their hands were then placed in two different positions during the breast surface scan: (1) hands at their waist and arms in slight abduction, and then (2) with both hands behind their head. Adjustments to the positioning were made so the centre of the projected patterns was below the areola of the participant’s breast (see example in Supplementary Fig. S2c). The participant was asked to hold their breath approximately 5–7 s during each scan to reduce motion artifact. Overall, the scanning procedure took less than 15 min and the entire process, including research description and change of clothes, took 30 min. The results from each SLS were processed by the aforementioned software (HP 3D Scan Pro 5.4.0, HP Inc) and exported for post-processing (see Supplementary S1.5 and S1.6).

### Assessment of print quality of the breast phantom

The reconstructed CT models of both left and right breast phantoms were imported into the HP software (HP 3D Scan Pro 5.4.0) and the *Distance* tool was used to obtain measurements. As a reference, the printed breast phantoms were measured by a caliper. Measurements of the two methods were compared to the ones in the computer model to assess the print quality of the phantoms. The measurements include length and width of calibration bar, diameter of fiducial markers, and distances between different markers.

### Assessment of completeness of surface scans

One measure of system performance was the completeness of surface scans. An operator visually inspected the partial and stitched surface scans, and scans were rated as either “complete” or “incomplete”. Complete visualization consisted of the entire breast surface including lateral and inferior aspects. The presence of any missing areas merited an “incomplete” rating.

### Assessment of accuracy between models and scans

The accuracy of the system consisted of trueness and precision. Trueness was evaluated by comparing surface scan results to the reconstructed CT models (reference model). Precision was evaluated by repeating the surface scans of each phantom three times. As a reference, the 3D breast model that was originally employed to print the phantom was also compared to the 3D scans.

For all comparisons, the distances between the reconstructed scans and the reference model were computed after scan-model alignment with cloud processing software (CloudCompare 2.10, GNU General Public License software)^25^. An operator first imported the stitched surface scan and the CAD model into the software. The scans and model were registered by manually placing three to five points at the fiducial markers and the registration was refined by the ICP algorithm. After landmark registration of the scan-model pair, multiscale model to model cloud (M3C2) comparison was employed to compute the distances. The algorithm created user-defined cylindrical volumes around subsets of points that were oriented normal to the surface points of the model, and all the points contained within the cylinder were then used to calculate the distance between the scan and the model^26^. The mean distances between the scans and the model were computed by M3C2. The values of mean distances computed were then averaged based on three repeated measures to assess accuracy.

### Assessment of the accuracy of estimated volumes

The last measure of system performance was the precision of the estimated volume from the collected data. To account for the print error, the reconstructed CT phantom model was used as the reference model. The model and the scans were registered using the SLS software (HP 3D Scan Pro 5.4.0, HP Inc). The posterior wall was created to simulate the use of posterior breast wall for human data in “Assessment of completeness of surface scans” section. Volumes of the scan and CAD model were determined by the *Mass Property* tool. Each scan-model pair calculation was repeated three times for each phantom at each position. As a reference, the volume of the original CAD model was also determined by Rhino using the *Mass Property* tool.

In addition, the feasibility of the proposed volume analysis method was assessed by manually segmenting breast volumes from the results of human subjects. The partial surface scans were first stitched together as described in “Assessment of print quality of the breastphantom” section. Then the complete surface scan for each individual in each posture was imported into Rhino 6.0 (Robert McNeel & Associates, Seattle, WA, USA) to calculate breast volume as described in “Assessment of completeness of surface scans” section. A one-way ANOVA was used to determine the effect of body postures on breast volumes. A second one-way ANOVA was performed to evaluate the effect of body postures on completeness of the surface scans. Post-hoc analysis was conducted when differences were found in the ANOVA results.

## Supplementary information

Supplementary Information.
