# Supplementary material for: Structured-light surface scanning system to evaluate breast morphology in standing and supine positions
Source: Sci Rep. 2020 Aug 24;10:14087. doi: 10.1038/s41598-020-70476-2 (PMC7445296; doi:10.1038/s41598-020-70476-2)
Supplement: Supplementary file 1 — Supplementary Information. [file 41598_2020_70476_MOESM1_ESM.pdf]

Structured-light surface scanning system to evaluate breast morphology in standing and supine positions

Olivia L.H. Tong[a,b], Astrid Chamson-Reig[a], Lawrence C.M. Yip[a,c], Muriel Brackstone[d,e], Mamadou Diop[a,b,c], Jeffrey J.L. Carson[a,b,c,e,*]
[a]Imaging Program, Lawson Health Research Institute, 268 Grosvenor Street, London, Ontario, Canada, N6A 4V2; [b]School of Biomedical Engineering, The University of Western Ontario, 1151 Richmond Street, London, Ontario, Canada, N6A 3K7; [c]Department of Medical Biophysics, The University of Western Ontario; [d]London Regional Cancer Program, London Health Sciences Centre, 800 Commissioners Road East, London, Canada, N6A 5W9; [e]Department of Surgery, The University of Western Ontario

Supplementary Information

S1 Materials

S1.1 System setup

A schematic of the structured-light scanning (SLS) system is presented in Figure S1(a). The two scanners were mounted on an articulating arm with the ability to switch their orientation to scan both at the standing and supine positions [Figures S1(b) and S1(c)] from different angles. The articulating arm was attached to a wheeled medical cart (dimensions 52 cm x 92 cm x 64 cm) for mobility. Each SLS system consisted of two cameras (STC-MBS231U3V, Sentech America, USA) and a projector (K132, Acer Inc, Taiwan) as shown in Figure S1(a). Each camera was fitted with a fixed focal lens (M1214-MP2, 12 mm focal length, Computar, USA) and connected to a laptop (ThinkPad W540, Lenovo Group Ltd., China) via a USB 3.0 port.

Each projector was connected to the laptop and projected over an area of 550 mm x 340 mm at a working distance of 60 cm. The original HP cameras that came with the commercial package had a field of view (FOV) of 240 mm x 180 mm at a working distance of 60 cm. These cameras were replaced with the Sentech cameras which provided a FOV of 534 mm x 400 mm at the same distance. System control and image acquisition was performed using the software provided by HP.

S1.2 Dual color 3D-SI

While two sets of SLS systems improved system coverage, intersystem cross talk prevented these systems from operating simultaneously on the same field of view. To overcome this limitation, two optical filter sets were installed on the cameras and projectors [Figure S1(a)]. Blue and green filters were selected to minimize interference between scanners. The green

filter set was comprised of a high performance OD4 long-pass filter at 500 nm (TECHSPEC, Edmund Optics, Ø25 mm and Ø50 mm). The blue filter set was comprised of a high performance OD4 short-pass filter at 450 nm (TECHSPEC, Edmund Optics, Ø25 mm and Ø50 mm).

(a) (b) (c)

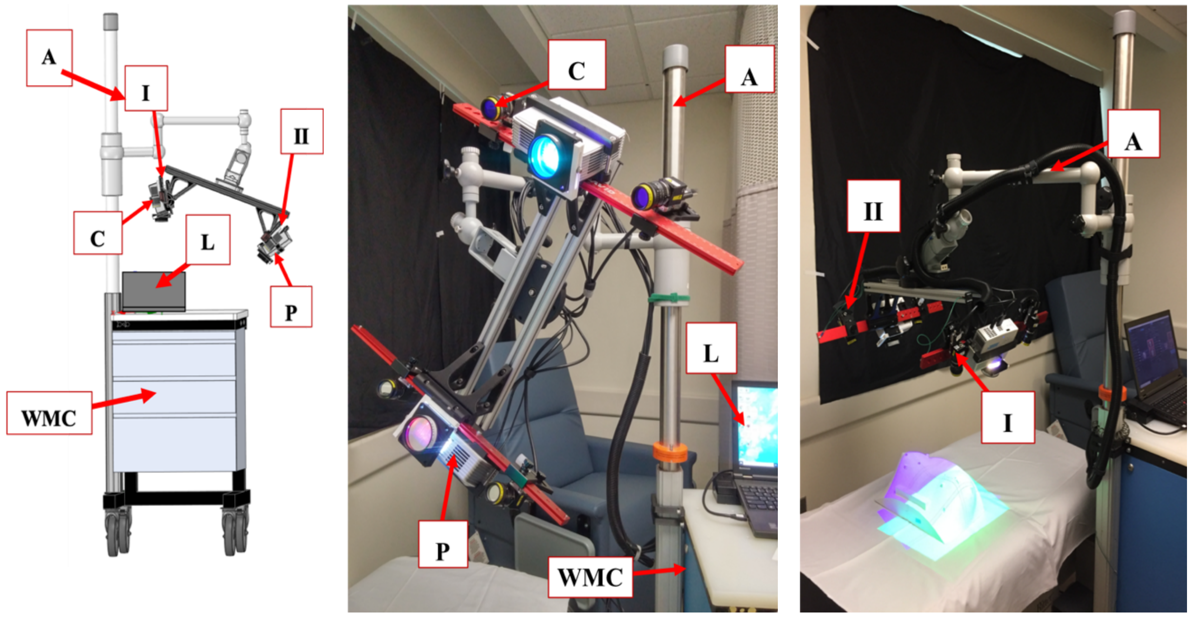

Figure S1. Setup of the 3D-SI system: (a) Schematic diagram of the set-up of the two sets of camera-projector systems. (b) Photograph of the 3D-SI system oriented for imaging the standing position. (c) Photograph of the 3D-SI system oriented for imaging the supine position. I – Camera-projector set with green filters. II – Camera-projector set with blue filters. A – Articulating arm. L – Laptop. WMC – Wheeled medical cart. C – Camera with filters. P – Projector with filters.

S1.3 3D printed breast phantom

A breast phantom was manufactured to evaluate the accuracy of the system. A computer model of a simulated human breast was designed, and 3D printed using white acrylonitrile butadiene styrene (ABS) filament. The phantom consisted of both left and right breasts, and each breast had a height of 214 mm and a width of 189 mm [Figure S2(a)]. Cylindrical fiducial markers with 5 mm radius were added to the model to facilitate scan alignment during post-processing of 3D scans.

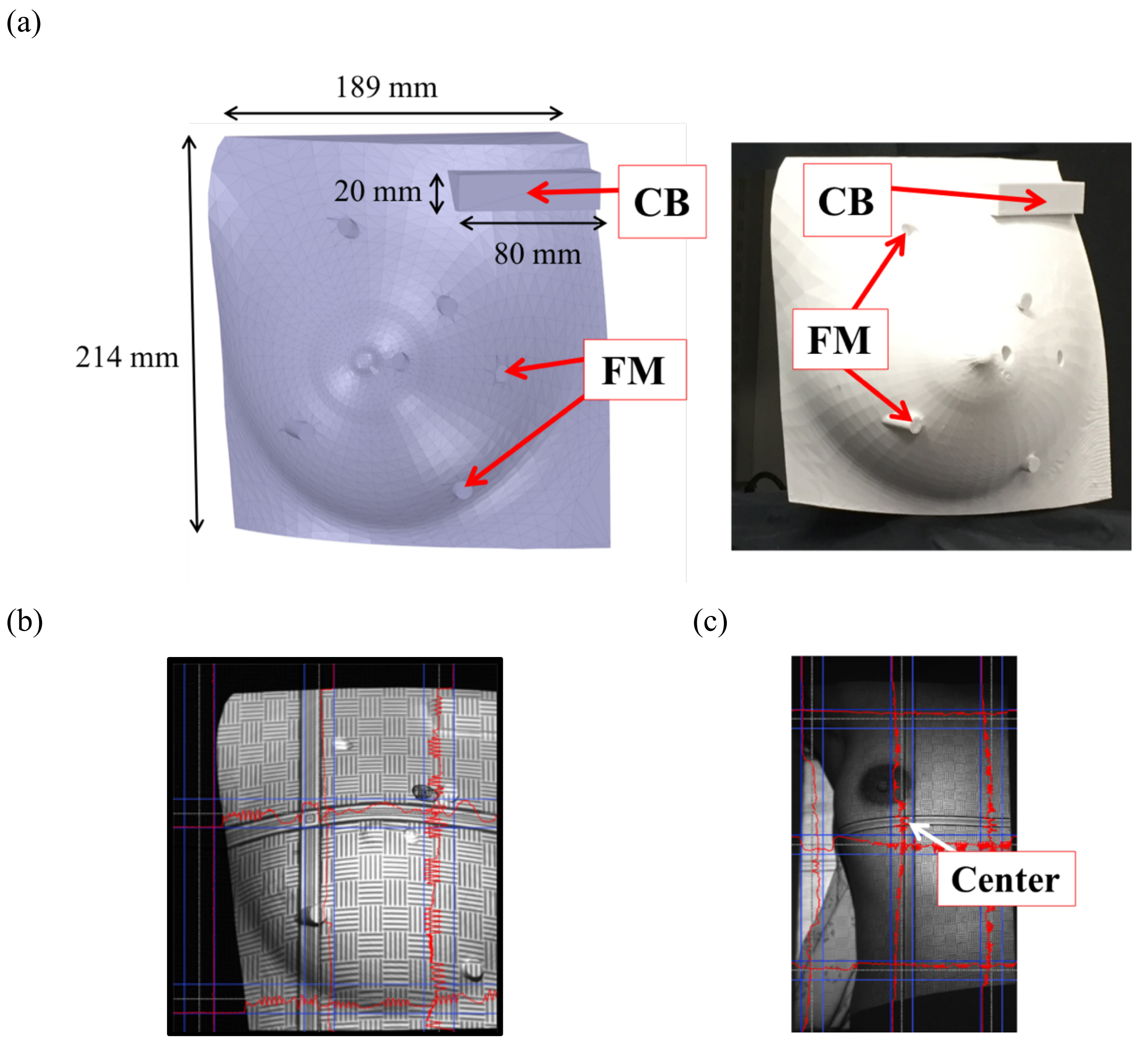

Figure S2. (a) Computer model (left) and photograph (right) of the 3D-printed breast phantom showing the right breast, calibration bars (CB), and fiducial markers (FM). Example view from one of the cameras with structured-light patterns (b) on the right breast phantom, and (c) on the right breast of a human participant in supine position.

S1.4 Human participants

Ten women were recruited for preliminary assessment of the 3D-SI system. Two parameters were reported: (i) bra size and (ii) breast ptosis. First, bra size is made up of cup size (in letter) and chest wall diameter (in number). Cup size is calculated by the difference between the fullest part of the breast and the chest circumference. Cup sizes of A and AA tend to have smaller breast volumes (50-250 cm^3), and cup sizes of D or more tend to have larger volumes (500 cm^3 or more). In addition to cup sizes, breast volume is also affected by chest diameter which is measured at the inframammary fold. In this paper, bra sizes were calculated by the method of Zheng et al. (2006). [27,28] Second, breast ptosis is a measurement to characterize breast

morphology. It is defined when the nipple drops to the level of the inframammary crease (IMC), and the classification of the degree of ptosis is adapted from the work of Kirwan (2002) [29]. Grade 0 (normal) is classified when the nipple position at least 1 cm above the IMC, Grade 1 ptosis is classified when the nipple position is even with IMC, and Grade 2 ptosis is classified when the nipple position is 1 cm below the IMC. The demographics of the female participants are listed in Table S1.

Table S1 Demographics of female participants

	Bra Size	Degree of ptosis
Subject 1	34B	Grade 0
Subject 2	42D	Grade 2
Subject 3	36D	Grade 1
Subject 4	30A	Grade 0
Subject 5	38A	Grade 0
Subject 6	36AA	Grade 0
Subject 7	32AA	Grade 0
Subject 8	36C	Grade 0
Subject 9	30A	Grade 0
Subject 10	40C	Grade 1

S1.5 Post-processing of surface scans: stitching

The 3D models from each SLS system were stitched together into a complete surface model by the HP software (HP 3D Scan Pro 5.4.0). The scans were coarsely registered using the fiducial markers, then finely registered by applying an iterative closest point (ICP) algorithm to optimize alignment.[30] The coarse and fine registrations were performed automatically using the fiducial markers between the scans from each SLS system. The registered scans were then exported and combined by merging common vertices of the scans in 3D mesh processing software (Meshlab 2016, GNU General Public License software)[31] to obtain a full mesh.

S1.6 Post-processing of surface scans: estimation of breast volume

The estimated volume of the breast depended on the extracted breast region; this region of interest was selected by examining the surrounding anatomical structures. We manually segmented the breast volumes using a breast volume analysis method modified from techniques described by Yip et al. (2012).[7] Briefly, the full mesh from each surface scan consisting of the breast and the surrounding anatomical structures as shown in Figure S3(a) was imported into a CAD software (Rhino 6.0, Robert McNeel & Associates, USA). A posterior breast wall was first estimated using a digital surface that followed the shape of the anterior chest curvature [Figure S3(b)]. Next, the breast surface that included the contour of the breast was extracted from the torso [Figure S3(c)]. The breast surface was then intersected with the posterior breast wall to form a solid, and the volume of the extracted region was obtained [Figure S3(d)]. Breast extraction was aided by the *Drape* tool, *Curve* tool, *Extrude* tool, *Patch* tool, and the *Boolean Split* tool within Rhino 6.0. The volume of the segmented breast was determined by the *Mass Property* tool within Rhino 6.0.

(a) (b) (c)

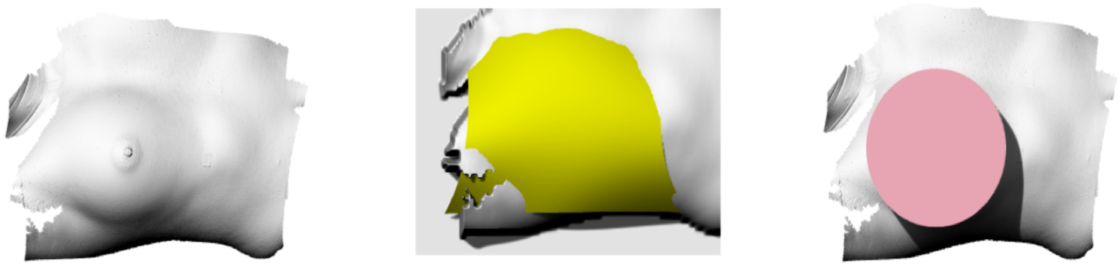

(d)

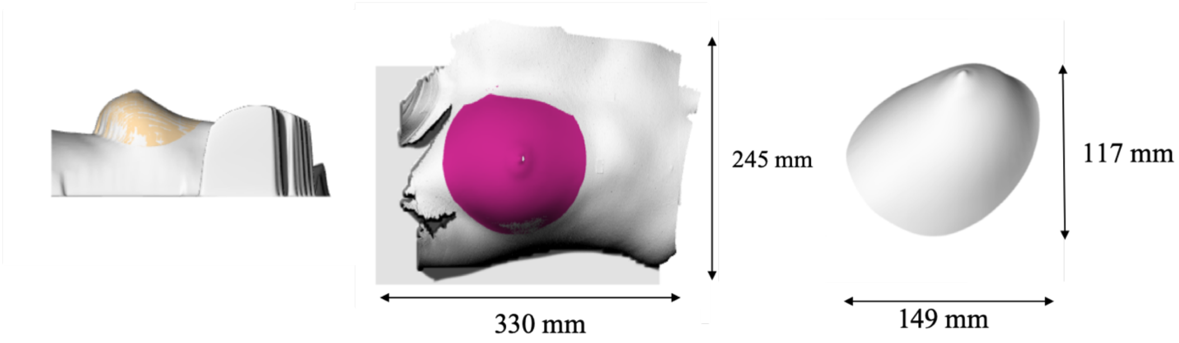

Figure S3. Breast volume analysis technique: (a) Example of an acquired breast surface scan imported into the CAD software. (b) Illustration of the digitally created posterior breast wall that followed the shape of the anterior chest curvature. (c) Example of breast surface extraction using an elliptic cylinder that included the breast border. (d) Illustration of the extracted breast region.

References

27. White, J. & Scurr, J. Evaluation of professional bra fitting criteria for bra selection and fitting in the UK. *Ergonomics* **55**, 704–711 (2012).
28. Zheng, R., Yu, W. & Fan, J. Breast measurement and sizing. in *Innovation and Technology of Women's Intimate Apparel* 28–58 (Elsevier, 2006). doi:10.1016/B978-1-84569-046-5.50002-1
29. Kirwan, L. A classification and algorithm for treatment of breast ptosis. *Aesthetic Surg. J.* **22**, 355–363 (2002).
30. David Group. *David SLS-1 Getting Started Guide* (David Vision Systems, Koblenz, Germany, 2012).
31. P. Cignoni, M. Callieri, M. Corsini, M. Dellepiane, F. Ganovelli, G. R. MeshLab: an Open-Source Mesh Processing Tool. in *Sixth Eurographics Italian Chapter Conference* 129–136 (2008).
